# In Vitro and Ex Vivo Evaluation of Rifampicin Cytotoxicity in Human Skin Models

**DOI:** 10.3390/antibiotics14070691

**Published:** 2025-07-08

**Authors:** Marcel Nani Leite, Natália Aparecida de Paula, Leandra Náira Zambelli Ramalho, Marco Andrey Cipriani Frade

**Affiliations:** 1 Division of Dermatology, Department of Internal Medicine, Ribeirão Preto Medical School, University of São Paulo, Ribeirão Preto 14040-900, São Paulo, Brazil; marcelnani@alumni.usp.br (M.N.L.); napaula@hcrp.usp.br (N.A.d.P.); 2 Department of Pathology and Legal Medicine, Ribeirão Preto Medical School, University of São Paulo, Ribeirão Preto 14040-900, São Paulo, Brazil; lramalho@fmrp.usp.br

**Keywords:** rifampicin, cell culture, skin explant model, toxicity, histological analysis

## Abstract

**Background/Objectives**: Drugs for human use require several studies for the assessment of their efficacy and safety. An important property is cytotoxicity, which should be tested in different environments and models in closer proximity to the final use of the drug, with greater reliability. Thus, we proposed to evaluate the toxicity of rifampicin, the only bactericidal drug in the anti-leprosy multidrug therapy, using skin cells and skin explant cultures. **Methods**: Cell viability was tested by the MTT method using primary keratinocytes and fibroblasts and immortalized skin cells (HaCaT and 3T3) at 24, 48, and 72 h of treatment. For the skin explant, we used the TTC assay to determine viability (24, 48, 72, and 96 h), hematoxylin and eosin staining to analyze the structure and architecture of the tissue, and TUNEL to assess apoptotic cells at 3, 6, 12, 24, 48, 72, and 96 h. **Results**: Regarding the toxicity of primary and immortalized cells, viability was above 70% up to a concentration of 50 μg/mL at 24, 48, and 72 h, and at the concentration of 200 μg/mL, all cells showed greater sensitivity, especially at 72 h. Tissue viability analysis revealed a high percentage (above 96%) of viable tissue at the concentrations of 100, 150, and 200 μg/mL at the time points studied. Histological analysis showed that tissue architecture was maintained, with no apoptotic cells being observed. **Conclusions**: Thus, our results showed the importance of evaluating drug toxicity using different cell types, with the ex vivo skin model proving to be an alternative to animal use.

## 1. Introduction

Preclinical experiments are necessary for the discovery and development of a drug [1] in order to evaluate some important aspects such as the toxicity, mutagenicity, teratogenicity, and carcinogenicity of the agent under study [2,3,4].

These initial steps require knowledge of physiology and a deep understanding of diseases, of the direct action of drugs, and of the effects of surgical interventions, among others. Ethical aspects related to the interventions are also important. Thus, experimental models become fundamental for a better understanding of natural phenomena [5].

A range of experimental models is described in the literature, varying according to the purpose of the study and the different scales used. They are divided into in vitro models that use cell cultures, ex vivo models using tissue and/or organ culture, computational models, alternative organisms such as Drosophila melanogaster, in vivo models using laboratory animals, and anatomical studies, usually conducted on cadavers [5,6,7,8].

The experiments begin with in vitro protocols, which are the use of cells extracted from both animals and humans and kept in the laboratory under specific conditions. Eukaryotic, bacterial, and yeast cells are used for experimental models that can assess biochemical, genetic, and molecular aspects, among others, for a better understanding of the various cellular events [1,9].

In addition to cell models, 3D models such as skin explants are also used for drug testing and can provide important results and information. They involve cultured fragments in their entirety or parts of them, representing the models closest to the in vivo model. All the tissue characteristics, as well as cells and structures, are maintained, and these explants can be cultivated for short or long periods ranging from days to months [6,10,11].

The human organotypic skin explant culture (hOSEC) represents an explant model that is closer to human skin under in vivo use conditions, with 3D dimensions, consisting of melanocytes, Langerhans cells, keratinocytes, dermal fibroblasts (glycosaminoglycans and collagen), and cutaneous appendages such as hair follicles and eccrine and apocrine sweat glands, with advantages over other existing alternative models [12,13]. This model is considered efficient for testing products that need to be evaluated for the efficiency of their activity and safety for human skin and has been consolidated as an alternative to the use of models involving experimental animals [14].

Rifampicin (RIF) is an antibiotic with amphoteric characteristics consisting of a red-orange powder with the molecular formula C_41_H_56_N_4_O_11_. Its pKa of 1.7 is related to the 4-hydroxyl group, while its pKa of 7.9 is related to the nitrogen of the piperazine group. It is slightly soluble in water, and its solubility and stability vary with pH due to its amphoteric nature [15]. It is a potent antibiotic of semi-synthetic origin of the rifamycin family, derived from rifamycin B, with broad-spectrum antimicrobial action, thus reaching both gram-positive and gram-negative bacteria and inhibiting the activity of the DNA-dependent RNA polymerase enzyme, thus suppressing bacterial RNA synthesis [16,17]. RIF was synthesized in the mid-1960s and subsequently added to the anti-leprosy treatment regimen, which was later labelled as multidrug therapy (MDT) by WHO [18] and is still used worldwide today [19,20].

The objective of the present study was to determine the toxicity of RIF to primary cells (keratinocytes and fibroblasts) and immortalized skin cells (HaCaT and 3T3), as well as in an alternative skin model (hOSEC), since no data about its action were found in the literature. We report here preliminary results obtained in these tests with RIF.

## 2. Results

### 2.1. Cell Viability

**Figure 1:**
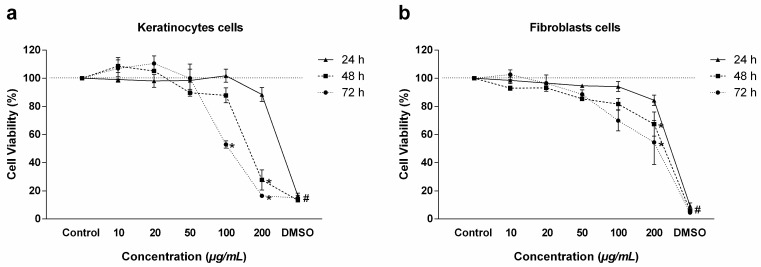
Viability of primary cells treated with RIF. (**a**) Viability of primary keratinocyte cells and (**b**) viability of primary fibroblasts at times of 24, 48, and 72 h as determined by the MTT assay. Values represent means ± SEM (n = 3). * corresponds to a significant difference compared to the control group. # corresponds to a significant difference between the DMSO group and the control group (*p* < 0.05) as determined by ANOVA followed by the Tukey post-test.

The MTT method was used to evaluate cell viability under exposure to RIF. Regarding the primary KC, high viability (above 88%) was observed at all concentrations studied (10, 20, 50, 100, and 200 µg/mL) within 24 h. Low viability of only 27% was observed within 48 h at the concentration of 200 µg/mL, and below 52% viability was also demonstrated both at the concentration of 100 µg/mL and at the concentration of 200 µg/mL within 72 h (Figure 1a). The same result was observed in FB within 24 h, with emphasis on viability above 84%. At 48 and 72 h, low viability of 67% within 48 h and of 54% within 72 h was observed only at the concentration of 200 µg/mL (Figure 1b).

**Figure 2:**
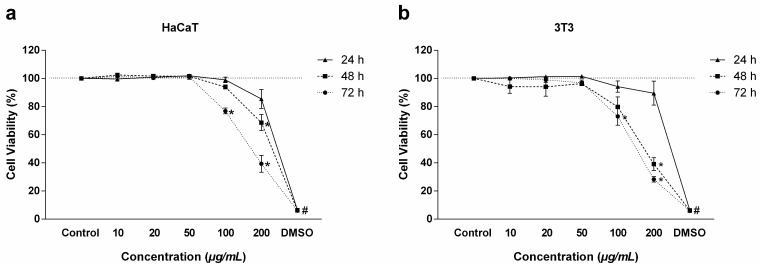
Viability of immortalized cells treated with RIF. (**a**) Viability of HaCaT cells and (**b**) viability of 3T3 cells at times of 24, 48, and 72 h as determined by the MTT assay. Values represent means ± SEM (n = 3). * corresponds to a significant difference compared to the control group. # corresponds to a significant difference between the DMSO group and the control group (*p* < 0.05) as determined by ANOVA followed by the Tukey post-test.

Regarding immortalized cells, the 24 h profile was practically the same for the HaCaT and 3T3 cell lines, with viability above 85% being obtained at all concentrations (Figure 2a,b). At 48 and 72 h, viability was below 68% only at the concentration of 200 µg/mL, with toxicity increasing in both cell lines, reaching 39% in HaCaT and 28% in 3T3 within 72 h (Figure 2a,b).

### 2.2. Tissue Viability

**Figure 3:**
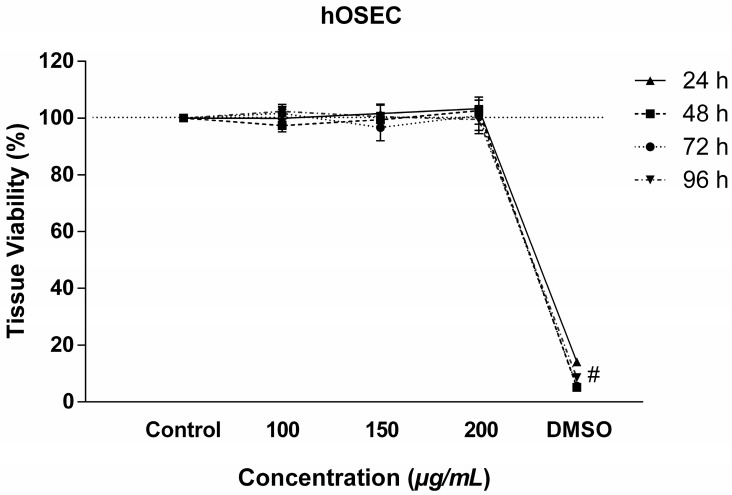
Viability of the hOSEC model treated with RIF. Viability of tissue at times of 24, 48, 72, and 96 h as determined by the TTC assay. Values represent means ± SEM (n = 3). # Corresponds to a significant difference between the DMSO group and the control group (*p* < 0.05) as determined by ANOVA followed by the Tukey post-test.

Tissue viability was assessed using the TTC method, the most effective test for this type of study. No decrease in viability was observed at the concentrations of 100, 150, and 200 µg/mL and at times of 24, 48, 72, and 96 h, with values above 96%, showing the safety of the concentrations used (Figure 3).

### 2.3. Histological Analyses and Apoptosis

**Figure 4:**
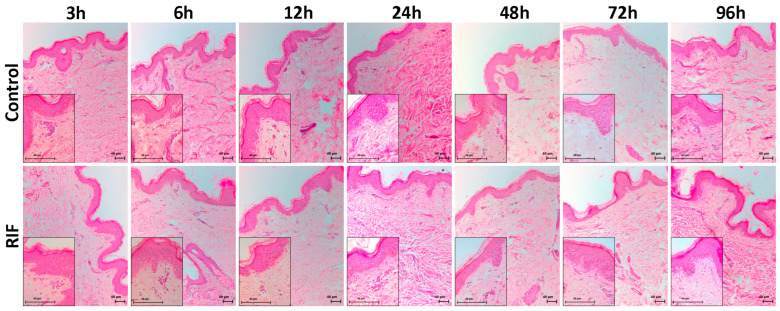
Histological analysis of the hOSEC model treated or not with RIF. Photomicrograph of skin stained with HE at times 3, 6, 12, 24, 48, and 72 h. (magnification: 100× and 400×).

HE staining was used for histological analysis, and no changes in tissue structure were observed at the times studied (3, 6, 12, 24, 48, 72, and 96 h) either in the presence or absence of RIF. Also, no necrotic cells or pyknotic nuclei were detected. Both the epidermal and dermal layers (papillary and reticular) were preserved in terms of cellularity, fibers, and attachments, and no changes in the dermo-epidermal junction were observed (Figure 4).

**Figure 5:**
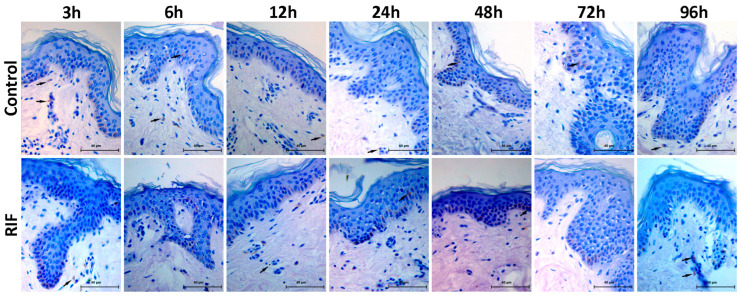
Apoptosis analysis of the hOSEC model treated or not with RIF. Photomicrograph of skin stained with the TUNEL assay at times 3, 6, 12, 24, 48, and 72 h. The black arrows indicate apoptotic cells (magnification: 400×).

Apoptosis was analyzed by the TUNEL method, which labels the cell nucleus in the apoptotic stage. Few cells in apoptosis were observed in the epidermis and dermis of the two groups at the times studied, with no difference between control and RIF treatment (Figure 5).

## 3. Discussion

The first step when testing any molecule or drug should consist of preclinical experiments that normally use cell monolayers. The cells are chosen according to the disease or target organ of interest [1,21,22]. In addition to cells, 3D models are also used, such as skin explants that can give satisfactory results, reducing or excluding the use of animals in research, besides providing greater complexity than monolayer cells, being closer to the tissue of origin, with a current worldwide trend regarding this aspect [13,23].

The results of these preclinical studies help us to find the best dose to be used and to determine the safety and the toxicity of the molecule or drug under study, as well as its true potential when using specific dosages according to the purpose of the work [24].

RIF has been used for a long time in the multidrug treatment of tuberculosis and is the backbone of the therapy. In addition, RIF has been used since 1981 in the treatment of leprosy as part of multidrug therapy together with two other drugs [25,26]. Approximately 85% of RIF is metabolized by microsomal enzymes, with the highest effects on the expression of cytochrome P450 (CYP) 3A4 in the liver and small intestine [27].

In view of the lack of literature data, we believed that it would be necessary to carry out toxicity tests of RIF applied to skin cells and directly to the skin. Thus, we conducted the present study testing the different models used by our work group. We decided to use both primary and immortalized skin cells in order to show the importance of each cell type. Primary cells are interesting because they are isolated directly from the target tissue and do not undergo any transformation or lack of control of the natural metabolism, showing behavior and characteristics closer to the natural ones of the host in addition to having all the natural characteristics of the tissue. However, their use is limited, and there may be differences among donor patients. Immortalized cells, on the other hand, undergo transformation so that they can be used in several passages, and their deviation is much smaller or almost none, thus permitting reproducible results [28,29]. In addition, they are commonly used as the gold standard for cytotoxicity assays. However, depending on the number of passages used, their phenotype, genotype, or morphology may change without perception, thus confounding the final result of the study [29,30,31,32].

Our results showed that there was no cytotoxicity to either primary or immortalized cells up to a concentration of 50 μg/mL, although at a concentration of 200 μg/mL viability was below 70% in KC, FH, and HaCaT within 72 h and in 3T3 within 48 and 72 h. No studies using these same cells to assess RIF toxicity were found in the literature; however, Singh et al. (2011) [33] used the HepG2 strain to assess the toxicity of RIF and showed that at concentrations of 25 μM (equivalent to 30.38 μg/mL), 50 μM (equivalent to 60.76 μg/mL), and 100 μM (equivalent to 121.51 μg/mL) for 24 h there was no cytotoxicity, results similar to ours. Another study has shown low macrophage cytotoxicity of RIF encapsulated in nanoparticles [34].

Our results regarding toxicity in the ex vivo skin model showed that concentrations of 100, 150, and 200 μg/mL at times of 24, 48, 72, and 96 h did not affect viability. No studies regarding cell viability in an ex vivo model using RIF are available in the literature. Vostálová et al. (2018) [35] also used a skin model, but without a drug, and showed that TTC is more effective than MTT and neutral red methods for toxicity testing.

RIF is a drug used for the treatment of both tuberculosis and leprosy, and cultivation of the leprosy bacillus is very difficult, with even greater difficulty in evaluating the toxicity of this and other drugs. Some tests were performed with macrophages infected with the bacillus, but this is still a difficult model to standardize [36]. Thus, the models most frequently used for drug evaluation are the in vivo models using mice. However, there is a delay in obtaining the results due to the slow multiplication of the bacillus [37]. Davis et al. (2013) [38] used mice infected with M. leprae treated with 10 mg/kg RIF for four weeks after 18 weeks of infection and showed that viability was reduced to 60%. Thus, the hOSEC model can become an effective and fast tool for the assessment of the toxicity and effectiveness of drugs against the leprosy bacillus.

In the present investigation, histological observations were an important parameter because tissue characteristics must be maintained during the toxicity study. Histology and TUNEL results showed that the skin structure remained viable and intact at a concentration of 200 μg/mL at times of 3, 6, 12, 24, 48, 72, and 96 h. There was no damage to the epidermis or dermis when RIF was applied compared to the control solution without RIF, although some apoptotic cells were observed in the epidermis and dermis both in the control group and in the group with RIF, but with no difference between them. The same model was used in another study by our group, showing that dacarbazine caused no damage to the skin during a period of up to 24 h [13]. Furthermore, the findings of Frade et al. (2015) [12] corroborate the maintenance of tissue viability in culture. Histomorphological analysis (HE staining) of the explants after 1, 7, 30, and 75 days of culture showed that, on the 7th day, the epidermis and the dermoepidermal junction were perfect.

## 4. Materials and Methods

### 4.1. Chemicals and Reagents

Rifampicin ≥ 95% (CAT #R3501), hydrochloric acid (CAT #100317), 1-(4,5-Dimethylthiazol-2-yl)-3,5-diphenylformazan (MTT) (CAT #M2003), 2,3,5-triphenyltetrazolium chloride (TTC) (CAT #17779), dimethyl sulfoxide (DMSO) (CAT #472301), ethanol (CAT #459844), and phosphate buffered saline (PBS) (CAT #P3813) were obtained from Sigma-Aldrich, St. Louis, MO, USA; Dulbecco’s modified eagle medium (DMEM) (CAT #12800017), defined keratinocyte medium (DKM) (CAT #10744019), fetal bovine serum (FBS), antibiotic and antimycotic (AA) (CAT #15240062) agents, and trypsin (CAT #15400054) were obtained from GIBCO—Invitrogen Corporation—Grand Island, NY, USA. Water was purified on a Milli-Q system (Millipore, Bedford, MA, USA).

### 4.2. Cell Lines

#### 4.2.1. Primary Cell Line

Human keratinocytes (KCs) and fibroblasts (FBs) were isolated from healthy skin fragments of patients undergoing abdominal plastic surgery according to Souto et al. (2006) [39]. Skin samples were stored overnight at 4 °C in a sterile flask containing PBS plus 1.5% AA. Excess adipose tissue was then removed, and the sample was cut into small pieces placed in 2 tubes of 15 mL containing 7 mL of 0.25% trypsin and incubated for 4 h at 37 °C in a humidified atmosphere containing 5% CO_2_. Trypsin was neutralized with 7 mL DMEM plus FBS, and the preparation was filtered through a 40 µm nylon filter and centrifuged at 1200 rpm for 10 min. Next, the supernatant was discarded, and the pellet was resuspended in 4 mL of culture medium specific for each cell type, placed in 25 cm^2^ bottles, and incubated at 37 °C in a humidified atmosphere containing 5% CO_2_. Keratinocytes were cultured in DKM and fibroblasts in high glucose (4.5 g/L) DMEM; both cultures were supplemented with 10% FBS and 1% AA, and the cells were used after 20 days of cultivation.

#### 4.2.2. Immortalized Cells

NIH-3T3 cells (a mouse fibroblast cell line) were purchased from the American Type Culture Collection (ATCC CRL-1658, Manassas, VA, USA), and HaCaT (human keratinocyte cell line) cells were acquired from the Cell Bank of Rio de Janeiro, RJ, Brazil. All cells were cultured in DMEM supplemented with 10% FBS and 1% AA and incubated at 37 °C in a humidified atmosphere containing 5% CO_2_.

### 4.3. Tissue Culture—hOSEC

Fragments of healthy skin were obtained from 18-to-50-year-old phototype II and III patients submitted to abdominoplasty. After surgery, the fragments were stored in a sterile and hermetically sealed bottle and transported to the Healing and Leprosy Laboratory of the Ribeirão Preto Medical School—USP. The fragments were incubated with PBS plus 1.5% AA overnight at 4 °C. The preparation was manipulated with sterile materials under laminar flow conditions, excess adipose tissue was removed, and fragments of 1 cm in diameter were sectioned with the aid of a histological punch. The fragments were placed on filter paper and a metal grid in a 12-well culture plate, one fragment per well, and were cultured with 2 mL DMEM culture medium supplemented with 10% (*v*/*v*) FBS and 1% (*v*/*v*) AA [13].

### 4.4. Drug and Dilution

A stock solution of 2 mg/mL RIF was prepared; the drug was first diluted in 50 µL DMSO, and the solution was then completed to 1 mL DMEM or DKM, filtered through a 0.22 µm filter, and protected from light. Next, dilutions of 10, 20, 50, 100, and 200 µg/mL were prepared for use in the cell viability assay. Concentrations of 100, 150, and 200 µg/mL were used in the tissue viability assay, and the concentration of 200 µg/mL was used for histological analysis and the determination of apoptosis.

### 4.5. Cell Viability Assay

The viability of cell lines was assessed using the MTT colorimetric method, which is based on the ability of living cell mitochondria to reduce the tetrazolium salt to purple-colored formazan crystals.

The cells were plated onto 96-well flat-bottomed microplates at a density of 2 × 10^4^ cells per well (200 µL volume) in triplicate for 24 h. Next, the volume was removed from the plate, and a solution of culture medium (respective cells) containing the different RIF concentrations (10, 20, 50, 100, and 200 µg/mL) was added. Culture medium (DMEM or DKM) without RIF and with 20% DMSO was used as a viability and cytotoxicity control, respectively. Cells were incubated at 37 °C in an incubator with 5% CO_2_, and viability was evaluated for 24, 48, and 72 h. After incubation, 20 µL of the concentrated MTT solution (5 mg/mL in PBS) with 180 µL of DMEM without phenol red or DKM was added to the cells, and the plates were incubated for 3 h. Then, 200 µL of DMSO was added to dissolve the formazan salts, and the reading was performed on a SpectraMax M3 microplate reader (Molecular Devices, CA, USA) at a wavelength of 570 nm. The result was expressed as a percentage of viability, which was determined by the relationship between the optical density (OD) of the different drug concentrations and the OD of the viability control, multiplied by 100 [40,41,42].

### 4.6. Tissue Viability Assay

Tissue viability was assessed using the TTC colorimetric method, which is based on the ability of live cells to reduce 2,3,5-triphenyltetrazolium chloride in insoluble formazan.

After mounting the plates with the skin, the culture medium was removed and DMEM+RIF was added at concentrations of 100, 150, and 200 µg/mL. DMEM alone was used as a viability control, and culture medium plus 20% DMSO was used as a cytotoxicity control. The plates were incubated at 37 °C in an oven with 5% CO_2_ for 24, 48, 72, and 96 h. After the incubation period, the solutions were removed, 1 mL of the 2% TTC solution prepared with DMEM, without phenol red, was added, and the plates were incubated under the same conditions as above for 2 h. The fragments were then washed with 0.9% saline solution and transferred to a new 24-well plate containing 1.5 mL DMSO/ethanol (1:1). Extraction was carried out overnight at room temperature in a plate shaker protected from light; 200 µL aliquots were then removed and transferred to a 96-well plate in triplicate, and a reading was obtained with a SpectraMax M3 microplate reader at a wavelength of 485 nm. The result was expressed as a percentage of viability, which was determined by the relationship between the optical density (OD) of the different drug concentrations and the OD of the viability control, multiplied by 100 [35,43].

### 4.7. Histological and Apoptosis Studies

After mounting the plates with the skin, the culture medium was removed and DMEM (control) or DMEM+RIF at the concentration of 200 µg/mL was added. The plates were incubated at 37 °C in an oven with 5% CO_2_ for 3, 6, 12, 24, 48, 72, and 96 h. After each incubation period, the samples were removed and placed in 10% formalin buffer (0.1 M phosphate buffer) for 24 h. The samples were then embedded in paraffin by the method of Andrade et al. (2017) [44] and stained with hematoxylin/eosin (HE). Apoptosis was determined by the TUNEL assay using the DeadEnd™ Colorimetric TUNEL kit (Promega Corporation, Madison, WI, USA) according to the manufacturer’s protocol.

A Leica DM 4000B^®^ light microscope equipped with a LEICA DFC^®^ 280 camera (Leica Microsystems, Wetzlar, Germany) was used to capture the histological images for both HE staining and the TUNEL assay. Images were taken at 100× and 400× magnification for HE staining and at 400× magnification for the TUNEL assay, using Leica Application Suite (LAS) software version 3.2.0.

### 4.8. Statistical Analysis

Data are reported as the mean ± standard error of means (SEM). Statistical variations between groups were determined by one-way ANOVA (variance for multiple comparisons) followed by the Tukey post-test. Values of *p* < 0.05 were considered significant. GraphPad Prism 8 software was used for all analyses (San Diego, CA, 176 EUA).

## 5. Conclusions

Based on these results, we may conclude that it is important to first evaluate drug toxicity in cell culture and at different concentrations, especially using cells from the tissue under study. The data also showed that both primary and immortalized cells can be used. We also show that rifampicin has greater toxicity in cell culture models, whereas it is nontoxic in the more complex skin model. In addition, the hOSEC model can be used as an alternative to the use of animals, thus being an important tool for obtaining other essential data.

## Data Availability

All data generated or analyzed during this study are included in this article. Further inquiries can be directed at the corresponding author.
